# Efficacy and safety of probiotics as adjuvant agents for *Helicobacter pylori* infection: A meta-analysis

**DOI:** 10.3892/etm.2015.2174

**Published:** 2015-01-09

**Authors:** ZHIFA LV, BEN WANG, XIAOJIANG ZHOU, FUCAI WANG, YONG XIE, HUILIE ZHENG, NONGHUA LV

**Affiliations:** 1 Department of Gastroenterology, The First Affiliated Hospital of Nanchang University, Nanchang, Jiangxi, P.R. China; 2 School of Public Health, Nanchang University, Nanchang, Jiangxi, P.R. China

**Keywords:** probiotics, *Helicobacter pylori*, adjuvant treatment, meta-analysis

## Abstract

The aim of the present study was to determine whether probiotics could help to improve the eradication rates and reduce the side effects associated with anti-*Helicobacter pylori* treatment, and to investigate the optimal time and duration of probiotic administration during the treatment, thus providing clinical practice guidelines for eradication success worldwide. By searching Pubmed, Embase, the Cochrane Central Register of Controlled Trials and the Science Citation Index, all the randomized controlled trials (RCTs) comparing probiotics as adjuvant agents of anti-*H. pylori* standard triple-therapy regimens with placebo or no treatment were selected. Statistical analysis was performed with the Comprehensive Meta Analysis Software. Subgroup, meta-regression and sensitivity analyses were also carried out. Twenty-one RCTs involving a total of 3,814 participants met the inclusion criteria. The pooled eradication rates of the probiotic group were 80.3% (1,709/2,128) by intention-to-treat (ITT) and 83.8% (1,709/2,039) by pro-protocol analyses; the pooled relative risk (RR) by ITT for probiotic supplementation versus treatment without probiotics was 1.12 [95% confidence interval (CI), 1.06–1.19]. A reduced risk of overall *H. pylori* therapy-related adverse effects was also found with probiotic supplementation (RR, 0.60; 95% CI, 0.40–0.91). The subgroup analyses showed that probiotic supplementation prior and subsequent to the treatment regimen both improved eradication rates for *H. pylori* infection. Furthermore, probiotic treatment lasting >2 weeks and including *Lactobacillus* or multiple probiotic strains significantly enhanced the efficacy. In conclusion, supplementation with probiotics for *H. pylori* eradication may be effective in increasing eradication rates and decreasing therapy-related side effects. Probiotic administration prior or subsequent to therapy and for a duration of >2 weeks may increase the eradication efficacy.

## Introduction

*Helicobacter pylori* is a Gram-negative spiral bacterium that colonizes the gastric mucosa. *H. pylori infection* affects 70–90% the population in developing countries, and 25–50% of the population in developed countries (1). Standard triple-therapy regimens with a proton-pump inhibitor (PPI) and two of amoxicillin, clarithromycin and nitroimidazole have been used for the eradication of *H. pylori*; however, their efficacy has been declining with the increasing resistance of *H. pylori* to antibiotics, and the *H. pylori* eradication failure rate varies widely, from 10 to 45% (2). At present, the administration of antibiotics for 10–14 days or high-dose PPI (twice a day) has been recommended for *H. pylori* eradication therapy by the Maastricht IV consensus conference (3); this has resulted in the increased incidence of undesirable side effects, such as antibiotic-associated diarrhea, nausea or vomiting, during anti-*H. pylori* therapy, which can lead to reduced compliance (4). Among the alternative anti-*H. pylori* options that have been considered, probiotics have attracted substantial interest. Previous studies have shown that probiotics*,* predominantly including *Lactobacillus*, *Saccharomyces boulardii* and *Bifidobacterium*, demonstrate anti-*H. pylori* activity *in vitro* and in animal models of *H. pylori* infection (5–8). Probiotics have also been used as an adjuvant therapy to *H. pylori* infection in order to reduce the side effects of antibiotics and improve the eradication rates (9–11); however, the results have been inconsistent, with certain studies showing that adjuvant probiotics did not improve eradication rates or reduce the side effects (12–14).

Previous meta-analyses have demonstrated that probiotics, as adjuvant agents, have a positive effect on improving eradication rates and reducing adverse events (15–20); however, certain recent studies have produced results that are inconsistent with those of the previous meta-analyses (21,22). Furthermore, the appropriate timing and duration of probiotic administration are indeterminate (23,24). Miscellaneous probiotics may be used in an anti-*H. pylori* treatment regimen, but it is unclear whether the efficacy of different probiotics is similar. As such, an updated meta-analysis of randomized controlled trials (RCTs) comparing the eradication rates and adverse events of probiotics as an adjuvant treatment with those of a placebo (or blank control) in participants with *H. pylori* infection is required. The aim of the present study was to evaluate, by meta-analysis, the efficacy and safety of the administration of probiotics as adjuvant agents of standard triple-therapy regimens for *H. pylori* infection, and to investigate the appropriate timing and duration of the probiotic administration in order to provide evidence to support this use of probiotics in clinical practice.

## Materials and methods

### Study sources and search methods

The present meta-analysis was developed according to the Preferred Reporting Items for Systematic Reviews and Meta-Analyses statement guidelines (25). Pubmed (1966 to November 2013), Embase (1946 to November 2013), the Cochrane Central Register of Controlled Trials (Issue 11, 2013) and the Science Citation Index (SCI; 1945 to November 2013) were searched according to Medical Subject Heading and text terms: (*Helicobacter pylori* OR *H. pylori*) AND (probiotic OR probiotics OR yeast OR yeasts OR yogurt OR *Lactobacillus* OR *Bifidobacterium* OR *Saccharomyces*). Authors were also asked to provide unpublished randomized trial results. In addition, the ClinicalTrials.gov website (https://clinicaltrials.gov/) was searched for registered RCTs whose results had not yet been published, and relevant studies were identified from the references.

### Inclusion and exclusion criteria

Articles that were eligible for inclusion in the meta-analysis met the following inclusion criteria: i) RCTs; ii) any age, endoscopic findings and symptoms at the time of enrollment; iii) confirmation of eradication outcome by urea breath test, histology or *H. pylori* stool antigen ≥4 weeks after therapy; iv) trials comparing at least two branches of treatment consisting of a control group (with placebo or no additional intervention) and an experimental group (the standard triple-therapy regimen plus probiotics); v) restriction of the species of probiotics to *Lactobacillus, Bifidobacterium, Saccharomyces* or a mixture of the three; vi) obtainable eradication rates.

The exclusion criteria for the meta-analysis were as follows: i) Undeterminable eradication rates; ii) use of agents other than probiotics as the adjuvant therapy for *H. pylori* infection in the experimental group; iii) articles without full-text; iv) studies in languages other than English.

### Validity assessment

Two reviewers independently, but not blinded to the authors or journal, assessed the quality of the studies that met the inclusion criteria. Any disagreements between the reviewers were resolved by consulting a third reviewer. The quality of the studies was assessed by the Jadad scale (26,27). The scores, from 0 to 5, were evaluated according to three criteria: Randomization, double blinding and description of withdrawals and dropouts (26,27). To avoid the duplication of data, if trials were published repeatedly by the same authors or institutions, only the most recently published article was included.

### Data extraction

Standardized data abstraction sheets were prepared. Data were extracted for study quality and type; the timing of probiotic administration; duration of eradication treatment; duration of probiotic treatment; species of probiotics; location of trials; time of publication; anti-*H. pylori* regimens; number and age of enrolled patients; diagnostic methods for detecting *H. pylori* infection prior to enrollment and subsequent to study completion; eradication rates by intention-to-treat (ITT) analysis; rates of successful and failed eradication; and total side effects (diarrhea, vomiting nausea, taste disturbance, epigastric pain and total adverse effects) from all included studies.

### Statistical analysis

Statistical analysis was performed with the Comprehensive Meta-Analysis Software (version 2; Biostat, Inc., Englewood, NJ, USA). The primary outcomes for the meta-analysis were the *H. pylori* eradication rates and the side effects among the trials comparing probiotic and control arms, based on ITT and pro-protocol (PP) analysis. The efficacy of *H. pylori* eradication was measured using relative risk (RR) to compare the frequency of eradication in the probiotic arm with that in the control arm.

The RRs for all studies were pooled into a summary RR, using either a fixed- or random-effects model, based on inverse variance methods. If the heterogeneity had a statistically significant difference, the random-effects model was employed; if not, the fixed-effects model was adopted. P-values and 95% confidence intervals (CIs) were provided for the summary RRs. The heterogeneity index (I^2^) was additionally calculated. Other assessments of heterogeneity were accomplished using the Q-test, and a Z-test was employed to assess the pooled effects. Funnel plots, Egger’s test and Begg’s test were utilized to estimate the publication bias. Meta-regression analyses were performed to interpret the reasons for the heterogeneity.

### Subgroup analysis

Subgroup analysis for the meta-analysis was performed depending on the time that the probiotics were administered [‘before’ (used prior to the eradication regimens), ‘same’ (simultaneously with the eradication regimens) and ‘after’ (beginning with the eradication regimens and continuing subsequent to the eradication regimens)], the regimens utilized, the duration of the probiotic treatment (≤2 weeks and >2 weeks), the species of probiotics, the age of the subjects, the Jadad score (>2, and ≤2), the PPIs of the experimental group and the duration of the eradication regimens.

## Results

### Description of the studies

**Figure 1:**
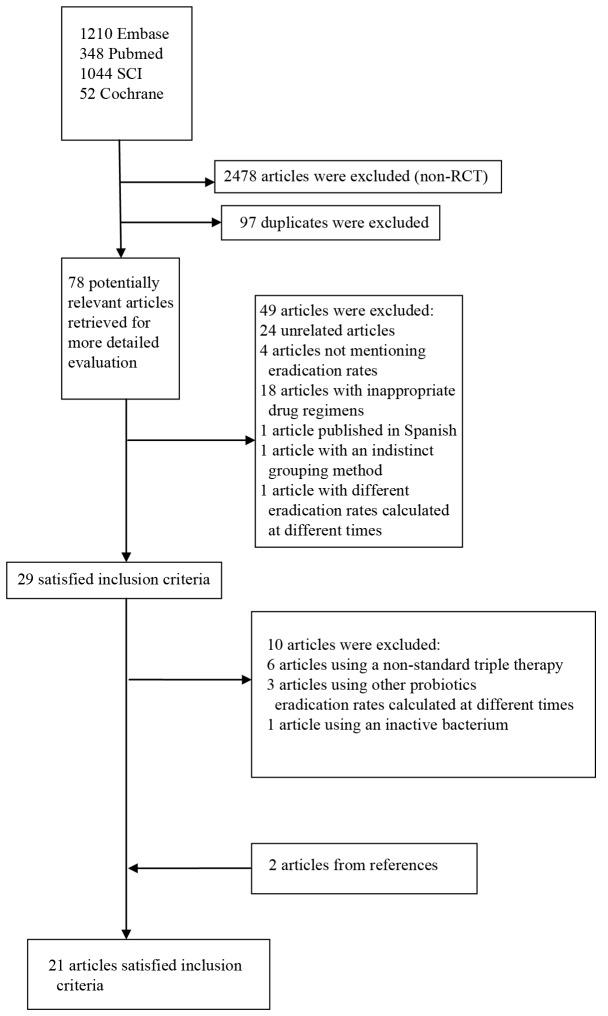
Flow diagram of the trials identified and selected. SCI, Science Citation Index; RCT, randomized controlled trial.

The bibliographical search yielded a total of 2,653 studies. Among the studies from Pubmed, the Cochrane Central Register of Controlled Trials, Embase and the SCI, another 2,478 articles were excluded subsequent to examining the article type. Having excluded any duplicates, 78 potentially relevant articles were retrieved for more detailed assessment. Following examinations of the title and abstract, another 24 unrelated articles, four articles that did not mention eradication rates, 18 articles with inappropriate drug regimens and one study published in Spanish (28) were excluded. The full-text articles were then reviewed and another two articles were excluded, one of which was excluded for indistinct grouping methods (29) and the other as a result of eradication rates being calculated at different times. Twenty-nine articles were further evaluated for details. Six articles were excluded due to a non-standard triple therapy regimen (9,12,30–33), three articles were excluded due to *Lactobacillus, Bifidobacterium or Saccharomyces* not being used in the eradication regimen (34–36) and one article due to an inactive bacterium being used (37). Two articles from the studied references were additionally included in the meta-analysis (38,39). Twenty-one RCTs ultimately met the inclusion criteria (11,38–57) (Table I) (Fig. 1).

Two institutions published two similar articles (40,41), in Italy. This triggered a concern about duplication of data; however, following a careful review of the articles in question, it was decided that the two articles were separate trials.

### Efficacy of H. pylori eradication

**Figure 2:**
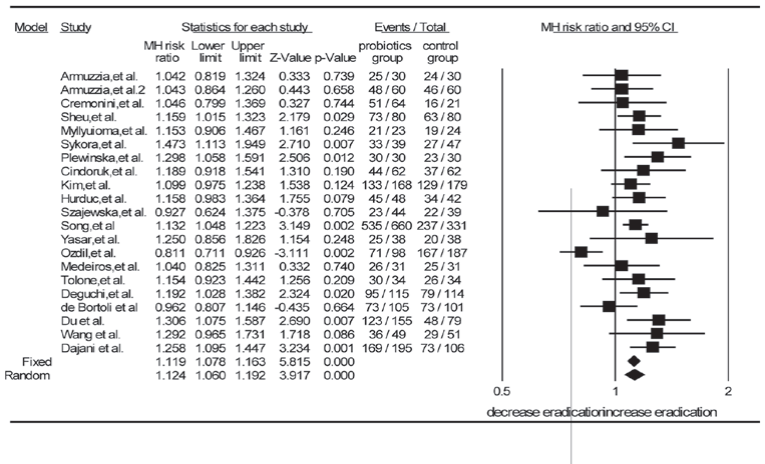
Forest plot comparing the eradication rate of supplementation by intention-to-treat analysis. CI, confidence interval; RR, relative risk; MH, Mantel-Haenszel.

The 21 RCTs included 3,814 patients in total, of whom 21 patients were in the probiotic group and 1,529 in the control group. The pooled eradication rate of the probiotic group was 80.3% (1,709/2,128) by intention-to-treat (ITT) and 83.8% (1,709/2,039) by PP analysis; the eradication rate in the probiotic group was higher than that in the control group (80.3 vs. 72.2%) with a statistically significant difference (Z=3.917, P<0.001). Using the random-effects model, the values of I^2^=52.3% and P=0.003 were obtained. The RR from a pooled analysis of the selected studies was 1.12 (95% CI, 1.06–1.19) by ITT analysis (Fig. 2).

### Subgroup analyses

Multiple subgroup analyses were carried out to explain the heterogeneity by stratifying the studies based on the timing of probiotic supplementation (‘before’, ‘same’ and ‘after’), eradication regimens, duration of probiotic supplementation, species of probiotics, age of patients (adults and children) and Jadad scores.

**Figure 3:**
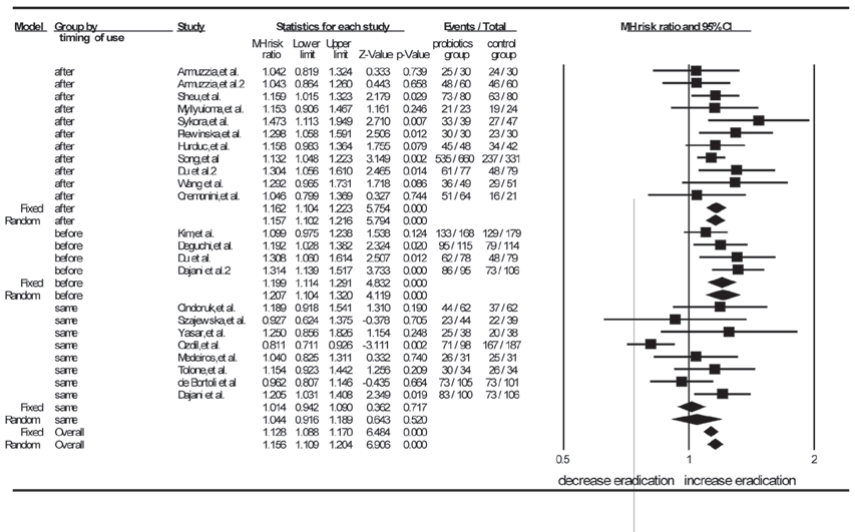
Forest plot of eradication rates grouped according to the timing of probiotic administration by intention-to-treat analysis. ‘Before’, probiotics were used prior to the eradication regimen and ended simultaneously the with regimen; ‘same’: probiotics were used and ended simultaneously with the eradication regimen; ‘after’, probiotics were used simultaneously with the eradication regimen and usage continued subsequent to the end of the regimen; CI, confidence interval; MH, Mantel-Haenszel.

There were 11 trials in which the probiotics were administered subsequent to the eradication regimens (38,40–46,50,52,56). The pooled analysis showed that the RR was 1.15 (95% CI, 1.10–1.21) according to the random-effects model. The RR of the eight studies in which probiotics were used simultaneously with the eradication regimens was 1.04 (95% CI, 0.92–1.19) by the random-effects mode (11,51,39,47,48,53,54,57). The RR for the probiotics used prior to the eradication regimens in the four RCTs was 1.21 (95% CI, 1.10–1.32) by the random-effects model (39,49,55,56). When the probiotics were administered prior or subsequent to the standard triple therapy, the differences between the experimental and control groups were statistically significant. When probiotics were used concurrently with the eradication regimens, no significant difference was found between the experimental group and the control group. This demonstrated that the timing of probiotic supplementation, i.e. prior or subsequent to the standard triple-therapy regimen, could improve the eradication rate (Fig. 3).

Subgroup analyses were additionally performed according to the eradication regimens (PPI plus amoxicillin and clarithromycin, PPI plus clarithromycin and tinidazole). The RRs analyzed in the random-effects model were 1.14 (95% CI, 1.07–1.21) and 1.04 (95% CI, 0.92–1.19) for the two regimens, respectively. The combination of the standard triple-therapy regimen (PPI, amoxicillin and clarithromycin) with probiotics achieved a significantly higher eradication rate than that obtained without probiotics; however, the combination of the PPI plus clarithromycin and tinidazole triple therapy with probiotics did not significantly improve the eradication rate for *H. pylori*.

According to the subgroup analyses of the duration of probiotic supplementation, the RR for durations of ≤2 weeks was 1.04 (95% CI, 0.98–1.11) by random-effects model, and the RR for durations of ≤2 weeks was 1.17 (95% CI, 1.12–1.23) by random-effects model. The results showed that probiotic supplementation for >2 weeks could improve the eradication of *H. pylori*.

According to subgroup analyses of species, the RR for *Lactobacillus* was 1.14 (95% CI, 1.08–1.25), the RR of *Saccharomyces boulardii* was 1.06 (95% CI, 0.89–1.23) and that of *Bifidobacterium* was 1.25 (95% CI, 0.86–1.82) (all by random-effects model). The RR in the multiple strains subgroup was 1.15 (95% CI, 1.08–1.22). The findings demonstrated that the use of *Lactobacillus* and multiple probiotic strains as adjuvant agents could improve the effectiveness of the *H. pylori* eradication to a greater extent than the control treatment; however, the administration of *Bifidobacterium* or *Saccharomyces boulardii* did not appear to improve eradication during anti-*H. pylori* treatment.

In the adult and children subgroup analyses, the RRs were 1.08 (95% CI, 1.01–1.16) and 1.22 (95% CI, 1.11–1.34), respectively, by the random-effects model. The results showed the enhanced efficacy of probiotic supplementation relative to that of the control treatment (P<0.001) in both adults and children.

There were 11 trials in which the Jadad scores were ≥3, indicating that their quality was high (40–42,44,45,48–52,56). The Jadad scores were <3 in 10 studies, which indicated that their quality was low (11,30,38,39,42,44–53, 56,57). According to the pooled analysis of the high-quality trials, the summary RR was 1.13 (95% CI, 1.08–1.19) by random-effects model. The RR of the studies of low quality was 1.12 (95% CI, 1.00–1.25) by random-effects model. The high-quality studies all showed the benefit of probiotic supplementation compared with the control treatment (P<0.001), but no significant difference was found between the treatments in the low-quality studies (P=0.053).

Subgroup analyses were also performed based on the different PPIs of the trial groups and the duration of the eradication regimens. In the omeprazole and rabeprazole subgroups, the differences between the probiotic and control groups were statistically significant (P<0.001 and P=0.034, respectively). Significant differences were also found for eradication durations of seven and 10 days (P<0.001 and P=0.012, respectively).

### Adverse events

**Figure 4:**
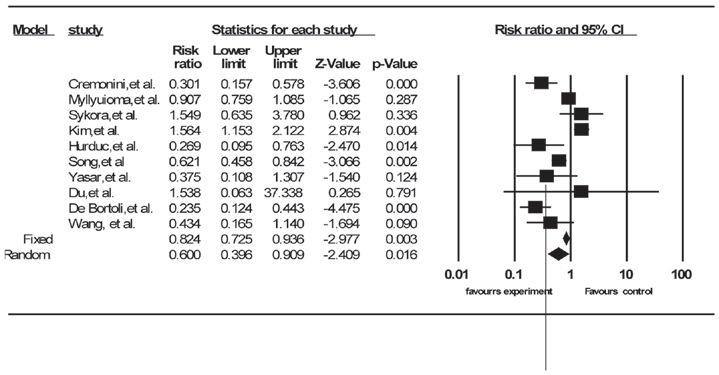
Effect of probiotic supplementation versus control treatment without probiotics on the incidence of total side effects. CI, confidence interval.

A total of 16 out of the 21 trials described side effects, including diarrhea, vomiting and nausea, and epigastric pain (30,38,39,42,44–53,56,57). Ten RCTs had data on total side effects (38,42,44,45,47,49,50,52,53,56). The summary RR was 0.60 (95% CI, 0.40–0.91) according to random-effects model analysis (I^2^=83.72%, P<0.001) (Fig. 4). Twelve RCTs reported the data for diarrhea (30,38,39,42,44,46–49,51,52,57), 10 for vomiting and nausea (30,38,42,44,46–49,52,57) and eight for epigastric pain (42,44,46–49,52,57). The pooled RRs were 0.42 for diarrhea (95% CI, 0.24–0.73) and 0.56 for vomiting and nausea (95% CI, 0.27–1.16) by random-effects model (I^2^=61.75%, P=0.002 and I^2^=60.05%, P=0.007, respectively), and 0.58 for epigastric pain (95% CI, 0.34–0.97) by the fixed-effects model (I^2^=34.00%, P=0.157). The majority of the studies did not provide details on how they estimated the severity of adverse events.

### Risk of bias in publication

**Figure 5:**
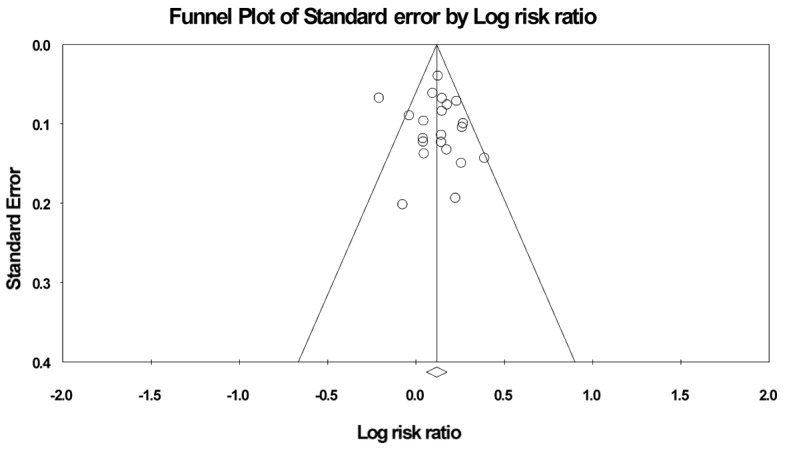
Funnel plot of included studies for eradication rates.

Funnel plot analyses by ITT analysis revealed slight asymmetry, but Egger’s test and Begg’s test showed no significant asymmetry of the funnel plot (Fig. 5).

### Sensitivity analysis

A sensitivity analysis was performed to interpret the reliability of the outcomes of the meta-analysis. Based on ITT analysis, the pooled RR values were established though the fixed- and random-effects models. The RRs were 1.12 (95% CI, 1.08–1.16) and 1.12 (95% CI, 1.06–1.19), respectively. No significant difference was found (overlapping CIs). When the largest study (52) was excluded from the sensitivity analysis, the RR did not change significantly (RR=1.12). The RRs were therefore steady.

### Heterogeneity

To interpret heterogeneity in the meta-analysis, a meta-regression analysis was performed. The results showed that the timing and duration of probiotic supplementation, the duration of the eradication regimen and the quality of study were the main causes of heterogeneity.

## Discussion

Probiotics, according to the World Health Organization, are defined as ‘live microorganisms, which, when administered in adequate amounts, confer a health benefit on the host’. They consist of bacteria and yeasts. It has been recognized that probiotics can exhibit an inhibitory ability against *H. pylori* (5). The effects of probiotics on *H. pylori* may be due to immunologic as well as non-immunologic mechanisms: i) Competition at the site of the stomach mucosal epithelium (6); ii) production of substances against *H. pylori*, such as acetic, propionic or butyric acid (58); iii) regulation of immune function and secretion of immunoglobulin A to improve mucosal defensive ability (59–61); and iv) strengthening tight junctions between epithelial cells (61,62).

The current results showed that probiotics could improve the eradication rate and decrease adverse events during anti-*H. pylori* treatment. The RRs were 1.12 (95% CI, 1.06–1.19) and 0.62 (95% CI, 0.40–0.91), respectively. The outcomes of the present meta-analysis were similar with several previous meta-analyses (15,17,18).

The optimal timing of probiotic administration is still unknown (23,24). It is generally believed that better efficacy occurs when probiotic supplementation occurs concurrently with or subsequent to antibiotic regimens. When probiotics are administered prior to antibiotic regimens, *H. pylori* is converted from a spiral to a coccoid form, which can lead to eradication failure. The timing of the addition of probiotics has been different in clinical trials (38,56,57). Whether the variability affected the eradication rate of *H. pylori* is not clear. The results of the current meta-analysis suggested that probiotic supplementation could improve eradication rates when provided prior or subsequent to the standard treatment regimens, but that supplementation supplied concurrently with the regimen did not significantly improve the eradication rate. The reason behind this may be that, when probiotics and antibiotics are administered simultaneously, it is inevitable that the antibiotics restrain the growth of the probiotics, resulting in a decrease in the anti-*H. pylori* substances produced by the probiotics (23,24).

An additional undetermined factor in studies to date is the appropriate duration of probiotic administration (23,24). In the present meta-analysis, the duration of probiotic administration varied from 7 days to months (38,45,47,51). The results suggested that a duration of >2 weeks could significantly improve the eradication rate for *H. pylori* infection, while a duration of ≤2 weeks could not. This indicated that the long-term administration of probiotics could be beneficial during anti-*H. pylori* treatment; however, further investigation is required to confirm this.

Based on the subgroup analyses of probiotic species employed, it was shown that the regimens with *Lactobacillus* were superior to the control group regimens (RR, 1.15; 95% CI, 1.05–1.25); however, only two RCTs used *Bifidobacterium* alone for adjuvant therapy during anti-*H. pylori* treatment. The effectiveness was slightly better than that of the control group regimens, but the difference was not statistically significant. Further investigation is therefore required to draw a definite conclusion. The use of *Saccharomyces boulardii* as a single supplement did not improve the eradication rate during anti-*H. pylori* treatment (RR, 1.05; 95% CI, 0.89–1.23). This suggests that the administration of *Saccharomyces boulardii* alone may not be suitable for adjuvant treatment during anti-*H. pylori* therapy (11,42,48,50,52).

Subgroup analyses were also performed according to different PPIs and durations of eradication regimens. It was of note that the omeprazole and rabeprazole subgroups achieved significant eradication success, while the esomeprazole, lansoprazole and pantoprazole subgroups did not, as compared with the control group. The current meta-analysis also demonstrated that the triple-therapy regimens with PPIs, amoxicillin and clarithromycin could achieve significantly higher eradication rates than the control group regimens, but the triple-therapy regimens consisting of PPIs, clarithromycin and nitroimidazole could not.

A number of studies have indicated that the administration of probiotics can ameliorate the symptoms and reduce the adverse effects associated with eradication therapy for *H. pylori,* such as diarrhea, vomiting, nausea and epigastric pain (14,35,47); however, certain investigations have suggested that probiotic supplementation does not reduce the incidence of side effects (21,49). The side effects experienced during anti-*H. pylori* regimens were therefore examined in the current meta-analysis, which showed that the supplementation of probiotics had a substantial effect on reducing *H. pylori* therapy-related adverse reactions, particularly diarrhea and epigastric pain. The results were consistent with those of previous meta-analyses (15,17,18). We believe that the application of probiotics has a beneficial effect and diminishes the discomfort during anti-*H. pylori* therapy.

To decrease bias in the present meta-analysis, the study selection, data extraction and assessment of study quality were performed by two reviewers. Another strength of the current meta-analysis was that it identified the majority of the RCTs published in English that used different species of probiotics as adjuvant agents for *H. pylori* treatment. The efficacy and safety of probiotics in anti-*H. pylori* treatment were comprehensively analyzed. The meta-regression analysis made the outcomes of the present meta-analysis reliable.

There were several limitations to the meta-analysis. Firstly, some evident heterogeneity was noted in the meta-analysis, although sub-analysis and meta-regression analysis were conducted to decrease the effects. Secondly, the language restriction could have influenced the results. There could have been a bias in the published languages, so it is likely that the present meta-analysis does not reflect all the outcomes of probiotics used for anti-*H. pylori* treatment. Finally, certain authors were asked for unpublished data, so the introduction of bias on that basis is possible. The Egger’s and Begg’s tests suggested that there could have been publication biases, and these could have affected the results of the meta-analysis.

In conclusion, the present meta-analysis showed that probiotic supplementation can improve eradication rates and reduce the adverse effects experienced during eradication therapy. In addition, probiotics appear to have enhanced effects on eradication rates when administered prior or subsequent to the standard regimens. Long-term probiotic treatment may have a superior effect to short-term probiotic administration. *Lactobacillus* and probiotic supplementation with multiple species appear to improve the eradication rate for *H. pylori* infection.

## Figures and Tables

**Table I:** Characteristics of the included studies.

First author, year (ref.)	Country, language	Total cases (treatment/control)	Patients	Eradication regimen	Regimen duration (days)	Species of probiotics	Time of probiotics^a^	Duration of probiotics (days)	*H. pylori* infection: Initial diagnosis re-checking	Jadad score
Armuzzia, 2001 (41)	Italy, English	120 (60/60)	Adults	P: 40 mg b.i.d. C: 500 mg, b.i.d. T: 500 mg b.i.d.	7	*Lactobacillus* GG	3	14	^13^C-UBT, *H. pylori* IgG antibody measurements/^13^C-UBT	3
Armuzzia, 2001 (40)	Italy, English	60 (30/30)	Adults	R: 20 mg b.i.d. C: 500 mg b.i.d. T: 500 mg b.i.d.	7	*Lactobacillus* GG	3	14	^13^C-UBT, *H. pylori* IgG antibody measurements/^13^C-UBT	5
Cremonini, 2002 (42)	Italy, English	85 (64/21)	Adults	R: 20 mg b.i.d. C: 500 mg b.i.d. T: 500 mg b.i.d.	7	*Lactobacillus* GG; *Saccharomyces boulardii*	3	14	^13^C-UBT/^13^C-UBT	5
Sheu, 2002 (43)	Taiwan, English	160 (80/80)	Adults	L: 30 mg b.i.d. A: 1 g, b.i.d. C: 500 mg, b.i.d.	7	*Lactobacillus*; *Bifidobacterium*	3	35	Histology and RUT/^13^C-UBT	2
Myllyuioma, 2005 (44)	Finland, English	47 (23/24)	Adults	L: 30 mg b.i.d. C: 500 mg b.i.d. A: 1 g b.i.d.	7	*Lactobacillus* GG (ATCC 53103); *L. rhamnosus* LC (DSM7061); *P. freudenreichii* ssp. *shermanii* JS (DSM7076); *B. breve* Bb99 (DSM 13692)	3	28	^13^C-UBT, serology/^13^C-UBT, serology [IgG decrease by 40% (four months)]	5
Sýkora, 2005 (45)	Czech Republic, English	86 (39/47)	Children	O: 10 mg (15–30 kg) or 20 mg (30 kg) b.i.d. A: 25 mg/kg b.i.d. C: 7.5 mg/kg b.i.d.	7	*Lactobacillus casei* (DN-114 001)	3	14	At least two of three: RUT, histology and culture/HpSAT, ^13^C-UBT	5
Płewinska, 2006 (46)	Poland, English	60 (30/30)	Children	O: 0.5 mg/kg/24 h, b.i.d. A: 50 mg/kg/24 h b.i.d. C: 15 mg/kg/24 h, b.i.d.	30	*Lactobacillus acidophilus*; *Lactobacillus rhamnosus*	3	28	RUT, histology/RUT, histology	1
de Bortoli, 2007 (47)	Italy, English	206 (105/101)	Adults	E: 20 mg b.i.d. C: 500 mg b.i.d. A: 1 g b.i.d.	7	*Lactobacillus reuteri*	1	7	HpSAT (99), ^13^C-UBT (107)/^13^C-UBT	2
Cindoruk, 2007 (48)	Turkey, English	124 (62/62)	Adults	L: 30 mg b.i.d. C: 500 mg b.i.d. A: 1000 mg b.i.d.	14	*Lactobacillus plantarum*; *L. reuterii*; *L. casei* subsp. *rhamnosus*; *Bifidobacterium infantis*; *B. longum*; *L. salivarius*; *L. acidophilus*; *Streptococcus termophilus*; *L. sporogenes* (Lactobacillaceae)	1	14	HE or Giemsa stain/^13^C-UBT	5
Kim, 2008 (49)	Korea, English	347 (168/179)	Adults	PPI b.i.d. C: 500 mg b.i.d. A: 1 g b.i.d.	7	*L. acidophilus* HY 2177; *L. casei* HY 2743; *B. longum* HY 8001; *S. thermophilus* B-1	2	28	RUT, ^13^C-UBT, histology/^13^C-UBT	3
Hurduc, 2009 (50)	Romania, English	90 (48/42)	Children	O/E: 1 mg/kg/day, b.i.d. A: 50 mg/kg/day, b.i.d. C: 15 mg/kg/day, b.i.d.	7 or 10	*Saccharomyces boulardii*	3	28	Histology, RUT/histology, RUT	3
Szajewska, 2009 (51)	Poland, English	83 (44/39)	Children	O: 0.5 mg/kg b.i.d. A: 25 mg/kg b.i.d. C: 10 mg/kg b.i.d.	7	*Lactobacillus* GG	1	7	Two of three tests (^13^C-UBT, histopathology, RUT)/^13^C-UBT	5
Song, 2010 (52)	Korea, English	991 (660/331)	Adults	O: 20 mg, b.i.d. A: 1 g, b.i.d. C: 500 mg, b.i.d.	7	*S. boulardii*	3	28	Histology/UBT	3
Yaşar, 2010 (53)	Turkey, English	76 (38/38)	Adults	P: 40 mg, b.i.d. A: 1 g b.i.d., C: 500 mg b.i.d.	14	*Bifidobacterium*	1	14	HE and modified Giemsa staining/^13^C-UBT	1
Medeiros, 2011 (54)	Portugal, English	62 (31/31)	Adults	E: 20 mg b.i.d. A: 1 g, b.i.d. C: 500 mg, b.i.d.	8	*L. acidophilus*	1	8	Culture/^13^C-UBT	2
Ozdil, 2011 (11)	Turkey, English	285 (98/187)	Adults	Group 1: L: 30 mg b.i.d. C: 500 mg b.i.d. A: 1 g b.i.d. Group 2: E: 40 mg b.i.d. Lev: 500 mg q.d. A: 1000mg b.i.d. Group3^b^: E: 40 mg b.i.d. A: 1000 mg b.i.d. for 5 days E: 40 mg b.i.d. L: 500 mg q.d. T: 500 mg t.i.d. for 5 days	14	*Saccharomyces boulardii*	1	14	Giemsa-staining/monoclonal HpSAT	1
Deguchi, 2012 (55)	Japan, English	229 (115/114)	Adults	R: 10 mg b.i.d. A: 750 mg b.i.d. C: 200 mg b.i.d.	7	*Lactobacillus gasseri* OLL2716	2	28	Culture, RUT, histology/^13^C-UBT, HpSAT or culture	2
Du, 2012 (56)	China, English	234 (155/79)	Adults	O: 20 mg b.i.d. C: 500 mg b.i.d. A: 1g b.i.d.	7	*Lactobacillus acidophilus*; *S. faecalis*; *B. subtilis*	2 or 3	21	RUT, ^13^C or ^14^C-UBT, pathology/^13^C or ^14^C-UBT	2
Tolone, 2012 (57)	Italy, English	68 (34/34)	Children	O: 1 mg/kg (before breakfast) b.i.d. A: 50 mg/kg (after meals), b.i.d. C: 15 mg/kg (after meals) b.i.d.	7	*Lactobacillus plantarum*; *L. reuterii*; *L. casei*. subsp. *rhamnosus*; *Bifidobacterium infantis* ; *B. longum*; *L. salivarius*; *L. acidophilus*; *Streptococcus termophilus*; *L. sporogenes*	1	7	^13^C-UBT/^13^C-UBT	2
Wang, 2014 (38)	China, English	100 (49/51)	Children	PPI: 0.6–0.8 mg/kg b.i.d. C: 10–15 mg/kg b.i.d. A: 30–50 mg/kg b.i.d.	14	*L. acidophilus*; *Bifidobacterium bifidum*	3	42	^13^C-UBT/^13^C-UBT	2
Dajani, 2013 (39)	Italy, English	301 (195/106)	Children	PPI: NR C: 500 mg b.i.d. A: 1000 mg b.i.d.	7	Bifidobacterium infantis	1 or 2	7 or 21	^14^C-UBT/^14^C-UBT	2

a 1 represents ‘same’, i.e. administration simultaneously with the eradication regimens; 2 represents ‘before’, i.e. used prior to the eradication regimen/continuing until the end of the eradication treatment; 3 represents ‘after’, i.e. beginning with the eradication treatment and continuing subsequent to the end of the eradication treatment/used when the eradication regimen has ended.

b Sequence therapy.

P, pantoprazole; C, clarithromycin; R, rabeprazole; T, tinidazole; L, lansoprazole; O, omeprazole; Lev, levofloxacin; E, esomeprazole; PPI, proton-pump inhibitor; b.i.d., twice a day; t.i.d., three times a day; q.d., daily; UBT, urea breath test; IgG, immunoglobulin G; RUT, rapid urease test; ATCC, American Type Culture Collection; HpSAT, *H. pylori* stool antigen test; HE, hematoxylin and eosin; NR, no report.
